# Intramyocellular lipid droplet–mitochondria uncoupling drives muscle atrophy via metabolite-directed epigenetic reprogramming: a review

**DOI:** 10.3389/fendo.2026.1980958

**Published:** 2026-10-13

**Authors:** Lingfeng Wu, Ye Wang, Kailian Ma, Yang Liu

**Affiliations:** 1 School of Acupuncture-Moxibustion and Tuina, Changchun University of Chinese Medicine, Changchun, Jilin, China; 2 Department of Pain Management, Affiliated Hospital of Changchun University of Chinese Medicine, Changchun, Jilin, China; 3 Department of Nephrology, Affiliated Hospital of Changchun University of Chinese Medicine, Changchun, Jilin, China; 4 Department of Acupuncture and Moxibustion, Affiliated Hospital of Changchun University of Chinese Medicine, Changchun, Jilin, China

**Keywords:** epigenetic reprogramming, lipid droplet-mitochondria contact, metabolic overflow, sarcopenia, type 2 diabetes mellitus, ubiquitin-proteasome system

## Abstract

Type 2 diabetes mellitus (T2DM)-associated sarcopenia is driven by protein degradation and impaired myogenic regeneration. While classical models emphasize cytosolic kinase pathways, the subcellular mechanisms mediating lipotoxicity-induced chronic catabolism remain incompletely understood. This review elucidates a tripartite pathological cascade comprising organelle structural uncoupling, metabolic intermediate overflow, and epigenetic reprogramming. Chronic intramyocellular lipid accumulation disrupts lipid droplet–mitochondria contact (LDMC) sites, largely via Mfn2 modification. This spatial dissociation impairs mitochondrial oxidation, driving the nucleocytoplasmic accumulation of polar metabolites, specifically Acetyl-CoA, succinate, and lactate. Acting as covalent substrates or competitive inhibitors, these metabolites modulate histone-modifying enzymes such as p300/CBP and JmjC-domain demethylases. Consequently, this substrate-driven epigenetic remodeling increases chromatin accessibility at E3 ubiquitin ligase promoters (*Atrogin-1*, *MuRF1*) and concurrently represses myogenic loci (*MyoD*, *MyoG*), locking the tissue in a catabolic transcriptional state. Effective interventions must target this organelle-metabolite-epigenetic axis. Beyond systemic agents like SGLT2 inhibitors and GLP-1 receptor agonists, precision strategies—including repairing LDMC topology, bypassing enzymatic bottlenecks via CPT1 or LDHA, and restoring epigenetic homeostasis—are necessary to preserve lean mass and maintain skeletal muscle under metabolic stress.

## 1 Introduction

### 1.1 Lipid metabolic background of type 2 diabetes-associated sarcopenia

#### 1.1.1 Ectopic intramyocellular lipid deposition and muscle wasting

In the pathogenesis of type 2 diabetes mellitus (T2DM), systemic lipid alterations correlate with the ectopic flux of free fatty acids (FFAs) into skeletal muscle, a process associated with myofibrillar protein degradation (1). Clinical and epidemiological studies demonstrate a correlation between intramyocellular lipid accumulation and reduced cross-sectional muscle area (1, 2). Conversely, observations in endurance-trained cohorts (the “athlete’s paradox”) indicate that elevated intramyocellular triacylglycerol (TAG) pools do not necessarily drive insulin resistance or cellular alterations (3). This indicates that toxicity depends not on total lipid pool size, but on subcellular compartmentalization. Consequently, current models suggest myofibrillar degradation is driven by the accumulation of reactive lipid intermediates (DAGs and ceramides) resulting from the structural uncoupling of organelle homeostasis (4, 5).

#### 1.1.2 upstream pathological cascades of intramyocellular lipid ectopic deposition

The etiology of IMCL ectopic deposition involves an alteration between transmembrane lipid influx and mitochondrial oxidative clearance. The translocation of the fatty acid translocase (FAT/CD36) increases the transmembrane influx rate of long-chain fatty acids (LCFAs) (6, 7). When the intracellular FFA substrate load exceeds the combined catalytic capacity of mitochondrial β-oxidation and the tricarboxylic acid (TCA) cycle, oxidation limitations emerge (8). This metabolic state increases the accumulation of unoxidized fatty acyl-CoAs, subsequently increasing DAG and ceramide concentrations at the inner leaflet of the sarcolemma. Preclinical models indicate that membrane-associated DAG activates novel protein kinase C isoforms (specifically PKCθ), which catalyzes the inhibitory phosphorylation of insulin receptor substrate-1 (IRS-1) at Ser1101, reducing the PI3K/Akt signal transduction cascade. Consequently, altered Akt kinase activity influences protein translation rates and contributes to the nuclear translocation of FoxO3, upregulating E3 ubiquitin ligases, notably *Atrogin-1*/*MAFbx* (9).

### 1.2 Limitations of cytosolic kinase and systemic models in lipid-induced muscle atrophy

#### 1.2.1 Insufficiency of linear signaling paradigms

Traditional mechanistic models characterize lipotoxicity as a product of systemic dysregulation or cytosolic kinase cascades. But drug treatments that only target these downstream signaling nodes show mixed results in keeping skeletal muscle mass in clinical trials, which means that upstream regulatory mechanisms are involved (10). Specifically, multi-omics analyses indicate that linear cytosolic signaling models do not fully account for the persistence of the catabolic state. For instance, *in vitro* C2C12 myotube models demonstrate that prolonged palmitate exposure drives atrophy independently of continuous upstream kinase activation (11). Concurrently, clinical biopsy data demonstrate that acute elevations in spatially restricted DAG pools induce localized insulin resistance in human skeletal muscle, independent of systemic shifts (12). Consequently, these observations indicate that lipotoxicity-induced muscle wasting extends beyond cytosolic kinase regulation. It involves the accumulation of intracellular lipid intermediates, which influences organelle interactions and alters chromatin-level epigenetic modifications (13, 14) (**Figure 1**).

**Figure 1:**
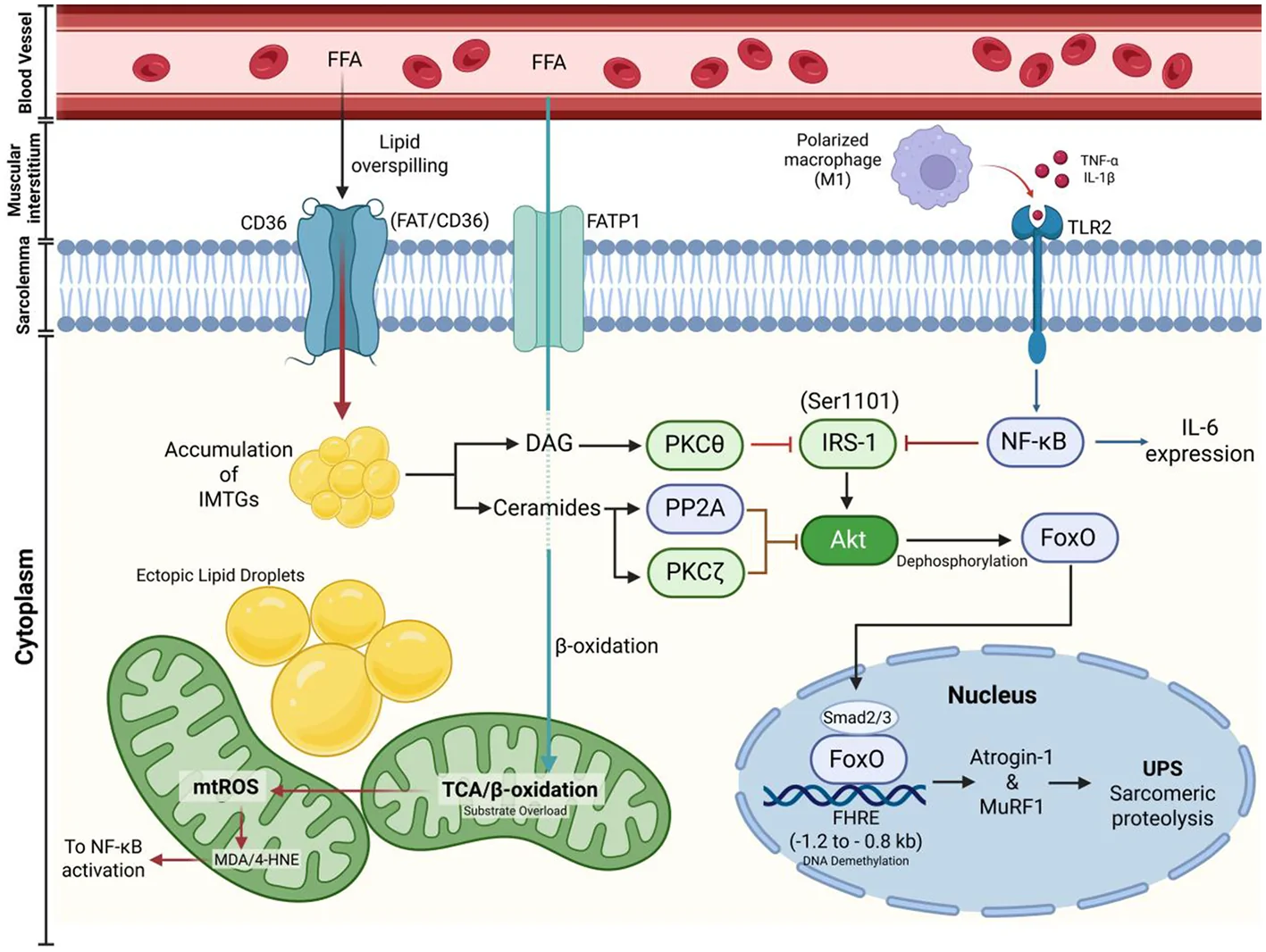
Canonical cytosolic signaling cascade of lipid-induced skeletal muscle atrophy. Systemic lipid overload facilitates the ectopic redistribution of free fatty acids (FFAs) into skeletal muscle via sarcolemmal transporters (CD36 and FATP1). The intracellular FFA burden exceeds mitochondrial oxidative capacity, leading to the accumulation of reactive intermediates, namely diacylglycerols (DAGs) and ceramides. DAGs activate PKCθ to phosphorylate IRS-1 at Ser1101, while ceramides simultaneously stimulate PP2A and PKCζ, collectively suppressing the PI3K/Akt signaling cascade. Attenuated Akt activity results in the dephosphorylation and subsequent nuclear translocation of FoxO3a, which, in conjunction with Smad2/3, binds to the Forkhead Response Elements (FHRE) at the promoters of atrophic E3 ubiquitin ligases (Atrogin-1 and MuRF1). Concurrently, mitochondrial substrate overload generates mtROS and lipid peroxidation products (specifically MDA and 4-HNE). These oxidative mediators, alongside M1 macrophage-derived cytokines (TNF-α and IL-1β) acting via TLR2, synergistically activate NF-κB-dependent inflammatory signaling (e.g., IL-6 expression). This integrated canonical pathway drives the ubiquitinproteasome system (UPS)-mediated degradation of sarcomeric proteins, culminating in skeletal muscle mass loss.

### 1.3 Core mechanistic hypothesis: the organelle-metabolite-epigenetic axis

This review explains a three-step mechanistic cascade: organelle changes, metabolite accumulation, and epigenetic modifications. In lipotoxic conditions, excessive FFA influx overwhelms mitochondrial oxidative capacity. This biochemical state is associated with Mfn2 degradation, which disrupts the specific Mfn2-Hsc70 tethering complexes required for lipid droplet-mitochondria contact (LDMC) sites (15). This spatial alteration disrupts substrate channeling, contributing to the accumulation of unoxidized lipid intermediates within the cytosolic compartment (16).

This loss of compartmentalization drives the nucleocytoplasmic translocation of these intermediates, altering the substrate pool for nuclear epigenetic enzymes. Subsequently, this nuclear lipid influx upregulates DNA methyltransferase 3B (*DNMT3B*) expression, contributing to non-CpG hypermethylation and altering chromatin accessibility at the *PGC-1α* promoter (17). Concurrently, organelle alterations influence the nucleocytoplasmic transport of the lipid droplet structural protein PLIN5, altering the catalytic balance between the deacetylase SIRT1 and the acetyltransferase GCN5. This kinetic shift destabilizes nuclear PGC-1α conformation (18, 19).

Consequently, site-specific epigenetic remodeling at the promoters of E3 ubiquitin ligases (*Atrogin-1*/*Fbxo32* and *MuRF1*/*Trim63*) increases local chromatin accessibility. This structural transition facilitates the binding of transcription factors (FoxO3 and Smad2/3) (20), directly upregulating ubiquitin-proteasome system (UPS) activity and driving sarcomeric protein degradation (10, 21, 22).

## 2 Lipid overload and disruption of organelle contact networks

Intracellular lipotoxicity correlates with the structural alteration of subcellular compartmentalization rather than uniform enzymatic degradation. Skeletal muscle lipid metabolism depends on a non-vesicular membrane contact network among specific organelles, modulating substrate transport, calcium flux, and redox equilibrium. Sustained lipid influx alters these physical contact zones, transitioning the local microenvironment from directed metabolic channeling to altered substrate diffusion. Consequently, this spatial alteration establishes conditions associated with intermediate metabolite accumulation and subsequent epigenetic modifications.

### 2.1 Physiological organelle tethering and lipid homeostasis

Skeletal muscle cells regulate intracellular lipid flux through an organelle contact network that integrates fatty acid transport, neutral lipid esterification, and mitochondrial oxidative phosphorylation. Within this topological framework, LDMCs and MAMs exhibit distinct functions: LDMCs participate in the transfer of lipid substrates toward mitochondrial oxidation, whereas MAMs regulate calcium ion transfer and endoplasmic reticulum (ER)-mitochondrial signal transduction.

Intramyocellular lipid droplets (LDs) and mitochondria form structural connections through membrane contact sites (MCSs) kept at a small 10–30 nm gap (23). High-resolution transmission electron microscopy (TEM) studies, which first defined the peridroplet mitochondria (PDM) subpopulation in brown adipocytes (24), and observations in skeletal muscle models (25), show that about 20% of the mitochondrial perimeter colocalizes with LDs within a <30 nm limit in oxidative myofibers. This arrangement is linked to the efficiency of lipid substrate utilization. LDs sequester excess free fatty acids into neutral triacylglycerol pools via esterification. Conversely, during elevated energetic demand, LDMCs facilitate the transfer of mobilized fatty acids into mitochondria for β-oxidation. This spatial compartmentalization dictates mitochondrial oxidative reaction rates in response to lipid influx, limiting the diffusion of lipid intermediates into the cytosol.

The stability of LDMC sites requires specific transmembrane tethering complexes. The lipid droplet surface protein Perilipin 5 (PLIN5) inserts its C-terminal hydrophobic helix directly into outer mitochondrial membrane (OMM) receptor assemblies (26). Furthermore, the SNARE protein SNAP23 and the GTPase Mfn2—which binds the LD-surface chaperone Hsc70 via its cytosolic domains—further stabilize this static organellar interface (15). Dynamically, this anchorage is modulated by kinase activity. AMPK-dependent phosphorylation stabilizes the GTP-bound conformation of Rab8a, promoting its assembly with PLIN5 (27). Upon energetic demand, PKA-mediated phosphorylation of PLIN5 at Ser155 induces a conformational shift that dissociates the coactivator CGI-58 to initiate triglyceride hydrolysis (28, 29). Following sustained metabolic stress, phosphorylated PLIN5 dissociates from the LD membrane and undergoes nucleocytoplasmic translocation, binding to PGC-1α to modulate transcription (18).

MCSs establish a microdomain that couples lipid mobilization kinetics to oxidative metabolism. The dissociation of CGI-58 from the Rab8a–PLIN5 complex recruits adipose triglyceride lipase (ATGL), initiating localized lipolysis at the contact interface (27). The interaction between PLIN5 and the OMM-bound fatty acid transport protein 4 (FATP4) couples triglyceride hydrolysis to the activation of acyl-CoA (29). By spatially confining free fatty acids within the <30 nm inter-organellar gap, this metabolic channeling prevents their escape into the broader cytosol, directly reducing the cytosolic accumulation of reactive intermediates such as diacylglycerols (DAGs) and ceramides (24).

At the endoplasmic reticulum (ER)-mitochondria interface, MAMs mediate local calcium ion flux and phospholipid exchange (30). This physical tethering enables the transport of calcium ions into the mitochondrial matrix, stimulating TCA cycle dehydrogenases to maintain respiratory chain electron flux under basal conditions (31). Consequently, MAMs integrate ER-localized lipid biosynthesis with mitochondrial oxidative phosphorylation. While deletion studies in specific myoblast models indicate that PLIN5-mediated physical coupling is dispensable for basal fatty acid oxidation rates (32), these MCSs correlate with augmented maximal respiratory capacity. This suggests that LDMC topological features primarily support metabolic efficiency during elevated substrate influx, rather than functioning as an obligate requirement for basal flux.

### 2.2 Lipotoxicity-induced contact site dissociation and uncoupling

Sustained lipid influx correlates with the structural dissociation of organellar interfaces (33). While direct topological evaluations in skeletal muscle are evolving, studies in hepatic and myocardial models indicate that elevated lipid concentrations structurally alter MAMs (34, 35) and Mfn2-dependent tethering complexes (15). These observations suggest a conserved lipotoxic mechanism where ER stress and targeted ubiquitin-proteasome degradation of Mfn2 reduce organellar coupling, reducing mitochondrial oxidative capacity and activating apoptotic pathways.

Confirming these cross-tissue observations within the specific context of skeletal muscle, high-resolution transmission electron microscopy demonstrates that excess lipid exposure reduces the frequency of these specialized membrane contact sites (MCSs), expanding the inter-organellar gap from a physiological <30 nm proximity to a spatially uncoupled state (>30 nm), concurrent with lipid droplet fusion and mitochondrial fragmentation (36). Mechanistically, this physical dissociation is linked to Mfn2 acetylation at Lys243 (K243), a modification that suppresses its GTPase activity and targets the protein for ubiquitin-proteasome degradation (15). The proteolytic clearance of Mfn2 dissociates the Mfn2–Hsc70 structural bridge. The transition from a <30 nm microdomain to a >30 nm gap alters lipid diffusion paths; the disruption of directional membrane-to-membrane transport directly increases the cytosolic accumulation of unoxidized reactive intermediates (37, 38).

The compartmentalization of lipid droplets additionally requires the surface expression of PLIN family proteins, including PLIN2 and PLIN5 (39). Under conditions of acute energy stress, AMPK activation phosphorylates PLIN2, targeting it for chaperone-mediated autophagy (40). However, in chronic lipotoxic environments where AMPK is typically suppressed, the accumulation of unesterified lipids and altered chaperone dynamics independently disrupt PLIN family stability, leading to PLIN2 displacement and subsequent lipid droplet fusion (diameters >2.0 μm) (41). At the same time, PLIN5 undergoes proteolytic cleavage, down regulating its protein expression (39), which severs the LD-mitochondrial tether and increases basal lipolysis rates.

These structural alterations precede specific enzymatic shifts. Higher free fatty acid influx and *de novo* lipid synthesis raise malonyl-CoA levels, which allosterically block CPT1, limiting long-chain fatty acyl-CoA from entering the mitochondrial matrix (42). At the same time, changed substrate flux causes a 3- to 5-fold accumulation of cytosolic long-chain acylcarnitines (11). When this excess lipid substrate is channeled into mitochondrial oxidation, electron flux overloads the electron transfer flavoprotein (ETF) system. This substrate overload hyper-reduces the ubiquinone pool, causing early electron leakage from respiratory Complexes I and III and creating mitochondrial reactive oxygen species (mtROS) (43). After this, cardiolipin oxidation and inner membrane depolarization further lower oxidative phosphorylation efficiency (44–46). As a result, the loss of LD-mitochondria spatial coupling, made worse by CPT1 inhibition, traps reactive lipid intermediates in the cytosol (32). This microenvironmental substrate overflow creates the biochemical base for downstream nuclear epigenetic modifications.

### 2.3 Structural uncoupling as a required step for metabolic problems

The structural separation of organelle networks happens before downstream metabolic blockages and intermediate accumulation. The loss of the LDMC limits the directed transfer of mobilized fatty acids for mitochondrial β-oxidation (24) Concurrently, increased *de novo* lipid synthesis elevates intracellular malonyl-CoA, which binds to the regulatory domain of carnitine palmitoyltransferase 1 (CPT1), inducing steric hindrance at its catalytic pocket and restricting long-chain fatty acyl-CoA (LCFA-CoA) entry into the matrix (42).

*In vitro* models utilizing lipid-overloaded C2C12 myotubes indicate that the overexpression of a malonyl-CoA-resistant CPT1 mutant (CPT1mt) maintains lipid oxidative flux and reduces palmitate-induced apoptosis (42). Furthermore, *in vivo* murine models utilizing CRISPR/Cas9-mediated skeletal muscle-specific knockout of carnitine palmitoyltransferase 2 (CPT2) demonstrate the accumulation of upstream lipid intermediates (11). Quantitative LC-MS/MS lipidomics reveal that CPT2 deficiency coupled with high-lipid exposure increases long-chain acylcarnitine species (C14:0, C16:0, and C18:1) by 3- to 5-fold, increasing their cytosolic abundance, which subsequently interferes with insulin receptor substrate (IRS) signal transduction (11). Consequently, the reduction in LDMC buffering capacity and the inhibition of mitochondrial oxidation enzymes increase the concentration of unoxidized lipids within the cytoplasm.

Alternatively, when excessive lipid influx overwhelms mitochondrial processing capacity despite CPT1 entry, the substrate load exceeds the catalytic limits of the TCA cycle and β-oxidation, resulting in incomplete oxidative reactions (8). This excess substrate alters the transfer of matrix-derived reducing equivalents (NADH and *FADH2*) to the respiratory chain. Higher lipid influx increases electron flux through the electron transfer flavoprotein (ETF) system, causing the over-reduction of the ubiquinone pool. This condition causes early electron leakage from respiratory chain Complex I (FMN binding site) and Complex III (Qo site) (43).

These uncoupled electrons reduce matrix oxygen to generate superoxides and hydrogen peroxide, which raises overall mitochondrial reactive oxygen species (mtROS) release rates (43). Ongoing mtROS production initiates lipid peroxidation, which oxidizes cardiolipin, a phospholipid needed for respiratory chain supercomplex assembly (45, 46). This structural change breaks down the transmembrane proton gradient, depolarizes the inner membrane potential (ΔΨm), and lowers oxidative phosphorylation (OXPHOS) efficiency, shown by a drop in the respiratory control ratio (RCR) to below 2.0 in isolated mitochondrial models (44). Additionally, elevated carbonyl species inhibit respiratory complex activities and correlate with cristae fragmentation (44). Specifically, the dissociation of LD–mitochondria spatial coupling limits the maximum respiratory capacity of the affected skeletal muscle cells (32). This mitochondrial metabolic stalling drives the nucleocytoplasmic efflux of unoxidized carbon intermediates, establishing the direct biochemical foundation for subsequent nuclear epigenetic remodeling.

## 3 Organelle uncoupling and the overflow of metabolic intermediates

The structural dissociation of organellar contact networks, particularly LDMCs, precedes specific biochemical alterations in skeletal muscle. This structural alteration limits directed metabolic channeling and increases the cytosolic diffusion of intermediate metabolites. Specifically, structural uncoupling correlates with specific enzymatic bottlenecks within mitochondrial β-oxidation and the tricarboxylic acid (TCA) cycle, altering the primary metabolic output of the mitochondria. The movement of polar metabolites (Acetyl-CoA, succinate, and lactate) across time and space from the mitochondrial matrix to the nucleoplasm provides direct structural substrates for downstream epigenetic modifications.

### 3.1 Acetyl-CoA accumulation: connecting fatty acid oxidation blockages and histone acetylation

The shift to epigenetic hyperacetylation is linked to a drop in mitochondrial substrate clearance (47). During high extracellular lipid exposure, extreme concentration gradients overcome normal LDMC structural limits, pushing the unchanneled, large movement of long-chain free fatty acids (LCFAs) into the mitochondrial matrix and causing a helpful but short-lived increase in β-oxidation flux (27). Each β-oxidation spiral makes one Acetyl-CoA molecule and equal amounts of reducing equivalents (NADH and *FADH2*) in the matrix (48). When substrate oxidation flux is more than the electron acceptance capacity of inner mitochondrial membrane (IMM) respiratory complexes I and II, the mitochondrial matrix redox state is changed (8). Biochemical tests using isolated mitochondrial preparations show that although the basal matrix NADH/NAD^+^ ratio stays near 0.1, experimental lipid overload raises this ratio to 0.5–1.0. This creates a reductive microenvironment when primary antioxidant defense networks like SOD and CAT become full (49).

This highly reduced state, marked by high NADH and proton motive force, helps reverse electron transport (RET). The increased reduction of the ubiquinone pool sends electron flow to the flavin mononucleotide (FMN) binding site of Complex I, causing RET and reducing molecular oxygen to make superoxide (O^2^·−) (50). This changed biochemical microenvironment blocks the TCA cycle. The catalytic activities of rate-limiting enzymes, especially isocitrate dehydrogenase (IDH3) and the α-ketoglutarate dehydrogenase complex (α-KGDH), depend on NAD^+^ availability. High matrix NADH lowers the activity of these dehydrogenases through direct product inhibition and allosteric modulation. This lowers their maximum reaction velocity (*Vmax*) and is linked to the accumulation of succinate and citrate (51).

At the same time, RET-derived lipid peroxidation products, like 4-hydroxynonenal (4-HNE), react through Michael addition with the free lipoic acid residues of the E2 subunit (dihydrolipoamide succinyltransferase) of α-KGDH. This covalent modification changes the enzyme’s conformation, lowering its catalytic velocity (52). Because the TCA cycle carbon consumption flux is lower than the continuous two-carbon input from β-oxidation, intermediate accumulation exerts negative feedback on thiolases and upstream components of the β-oxidation pathway. This sequential enzymatic inhibition reduces the decarboxylation rate of long- and medium-chain acyl-CoAs, mediating the accumulation of unoxidized lipid intermediates, specifically acylcarnitines (14).

Acetyl-CoA, the terminal product of β-oxidation, is restricted from crossing the IMM via passive diffusion due to the high polarity and molecular mass (~809 Da) of its coenzyme A moiety (53). During TCA cycle inhibition, Acetyl-CoA accumulates within the mitochondrial matrix, altering metabolic network equilibrium. Quantitative targeted metabolomics (LC-MS/MS) demonstrate that while basal Acetyl-CoA abundance in healthy muscle is 2.0–5.0 nmol/g wet tissue, lipid overload models correlate with a 2- to 3-fold increase in this local concentration (54).

While this accumulating matrix Acetyl-CoA pool concurrently activates pyruvate dehydrogenase kinase 4 (PDK4) to suppress aerobic glucose utilization via the Randle cycle (55), its primary consequence for epigenetic remodeling involves targeted structural export. To metabolize these trapped acetyl groups, carnitine acetyltransferase (CrAT) transfers acetyl moieties to free carnitine to yield acetylcarnitine, a metabolite possessing a dedicated transmembrane transporter (47). Because the equilibrium constant (*Keq*) of the carnitine acetyltransferase reaction is close to 1, the net direction of this reversible enzyme strictly follows matrix Acetyl-CoA concentration gradients. But, this specific substrate export pathway competes for the mitochondrial free carnitine pool, limiting its availability for CPT1-dependent transport and making lipid accumulation worse at the cytosolic leaflet (56).

When matrix Acetyl-CoA concentrations go past the processing capacity of CrAT, the higher substrate availability drives extensive lysine Nϵ-acetylation of mitochondrial proteins. Although possible mitochondrial acetyltransferases may take part, the weakly alkaline microenvironment of the matrix (pH ~7.8) naturally helps non-enzymatic chemical acetylation, where the thioester bonds of concentrated Acetyl-CoA go through direct nucleophilic attack by deprotonated lysine residues (47). This post-translational modification is kept going by the simultaneous drop of the deacetylase network. Specifically, NAD^+^ pool depletion lowers the catalytic activity of the NAD+-dependent mitochondrial deacetylase SIRT3 (57). The drop in SIRT3 activity keeps acetylation modifications on core metabolic enzymes, including long-chain acyl-CoA dehydrogenase (LCAD) (57). These covalent modifications change the spatial conformation of respiratory chain supercomplexes, lowering substrate affinity and leading to metabolic uncoupling (58). As a result, the continuous export of acetylcarnitine raises the pool of acetyl carbons in the cytosol and nucleus, giving substrates that help chromatin acetylation (53).

### 3.2 Succinate accumulation: connecting TCA cycle changes to epigenetic demethylation blockade

The accumulation of succinate connects tricarboxylic acid (TCA) cycle changes to the regulation of epigenetic states. In lipid overload, the structural separation of lipid droplet–mitochondria contacts (LDMCs) lowers oxidative flux, changing TCA cycle progression. This drop in enzymatic activity is linked to the accumulation of succinate in the mitochondrial matrix, where concentrations rise from a physiological baseline of ~0.2 nmol/mg protein to >1.5 nmol/mg protein (59).

Succinate shows structural homology with α-ketoglutarate (α-KG), the co-substrate for α-KG-dependent dioxygenases, like the TET protein family (TET1/2/3) and JmjC-domain histone demethylases, especially KDM5 and JMJD3. In basal conditions, these enzymes use α-KG and Fe^2+^ to activate molecular oxygen, making an oxoiron(IV) intermediate that helps the oxidative demethylation of methyl groups from DNA or histone tails (60). In lipid-exposed skeletal muscle, accumulated succinate acts as a structural analog. Due to this homology, succinate occupies the catalytic pockets of these dioxygenases, functioning as a competitive inhibitor (60).

*In vitro* kinetic assays demonstrate that succinate competitively inhibits JmjC family enzymes with a *Ki* of 350–500 μM (60), a concentration threshold exceeded by nuclear succinate levels during sustained lipid exposure. This active-site occupancy limits the assembly of the oxoiron(IV) intermediate, inhibiting the catalytic cycle and reducing DNA and histone demethylation rates (60). This biochemical inhibition is associated with DNA hypermethylation and the retention of repressive marks at specific regulatory zones, including bivalent promoters enriched with H3K27me3. The competitive inhibition of histone H3K27-specific demethylases, specifically KDM6A/JMJD3, maintains these responsive gene loci in a transcriptionally inactive state. Consequently, succinate accumulation resulting from TCA cycle inhibition provides a mechanism that alters chromatin accessibility and reduces myogenic differentiation (59, 60).

### 3.3 Lactate accumulation: connecting glycolytic shifts to histone lactylation

The accumulation of lactate during elevated lipid exposure correlates with mitochondrial oxidative alterations rather than a primary glycolytic defect. Structural alterations of LDMCs and the altered redox state of the mitochondrial matrix reduce TCA cycle and OXPHOS activity. This reduction in mitochondrial metabolism, coupled with PDK4-mediated PDH inhibition (55), strictly limits the mitochondrial uptake of cytosolic pyruvate. Concurrently, impaired mitochondrial electron shuttling causes NADH to accumulate within the cytosol, pathologically elevating the NADH/NAD^+^ ratio. To regenerate NAD^+^ required for basal cellular survival, this cytosolic retention of pyruvate and NADH forcefully drives the lactate dehydrogenase (LDHA) equilibrium toward lactate production. Therefore, rather than a programmed shift toward aerobic glycolysis, this process represents a compensatory mass-action reduction of pyruvate. While lactate is physiologically generated under aerobic conditions for intercellular shuttling (61), the lipotoxic mitochondrial gridlock severely distorts this equilibrium, trapping lactate as an accumulating cytosolic byproduct.

Preclinical NMR and LC-MS/MS metabolomics confirm this shift, indicating that while healthy skeletal muscle maintains a resting lactate concentration of approximately 1.5–2.0 mmol/kg wet weight (~15–20 nmol/mg protein), atrophic or high-fat-exposed murine muscle tissues exhibit concentrations exceeding 60–80 nmol/mg protein (54, 62, 63). This accumulation lowers intracellular pH and drives dual-compartment lactylation. Preclinical models indicate that hypoxia and lipid exposure initially induce the lactylation of mitochondrial proteins, which correlates with reduced catalytic efficiency of OXPHOS complexes (64).

At the same time, local accumulation helps lactate transport into the nucleoplasm. In the nucleus, lactate acts as a carbon donor for covalent histone modifications after its conversion into lactyl-CoA, which is then used by histone acetyltransferases, especially p300/CBP, for covalent modification on histone tails. Because of an extra hydroxyl group, the lactyl moiety brings a larger steric volume and higher polarity than acetyl groups. Lactylation of specific lysine residues causes electrostatic neutralization and steric hindrance, which changes the interaction between the histone octamer and the negatively charged DNA phosphate backbone, leading to an open, transcriptionally accessible chromatin conformation (65, 66).

Although this modification takes part in physiological responses, like MCT1-mediated lactate transport increasing TCA cycle flux during exercise (67), its ongoing rise is seen in lipid stress. Nuclear lactate gives a substrate for lactyl-CoA synthesis, which p300 uses to add histone H3K9la marks, controlling neuraminidase 2 (*Neu2*) transcription to affect myogenesis (68). In long-term lipid exposure, high histone lactylation changes chromatin accessibility, helping the transcription of atrophy-associated genes (**Figure 2**).

**Figure 2:**
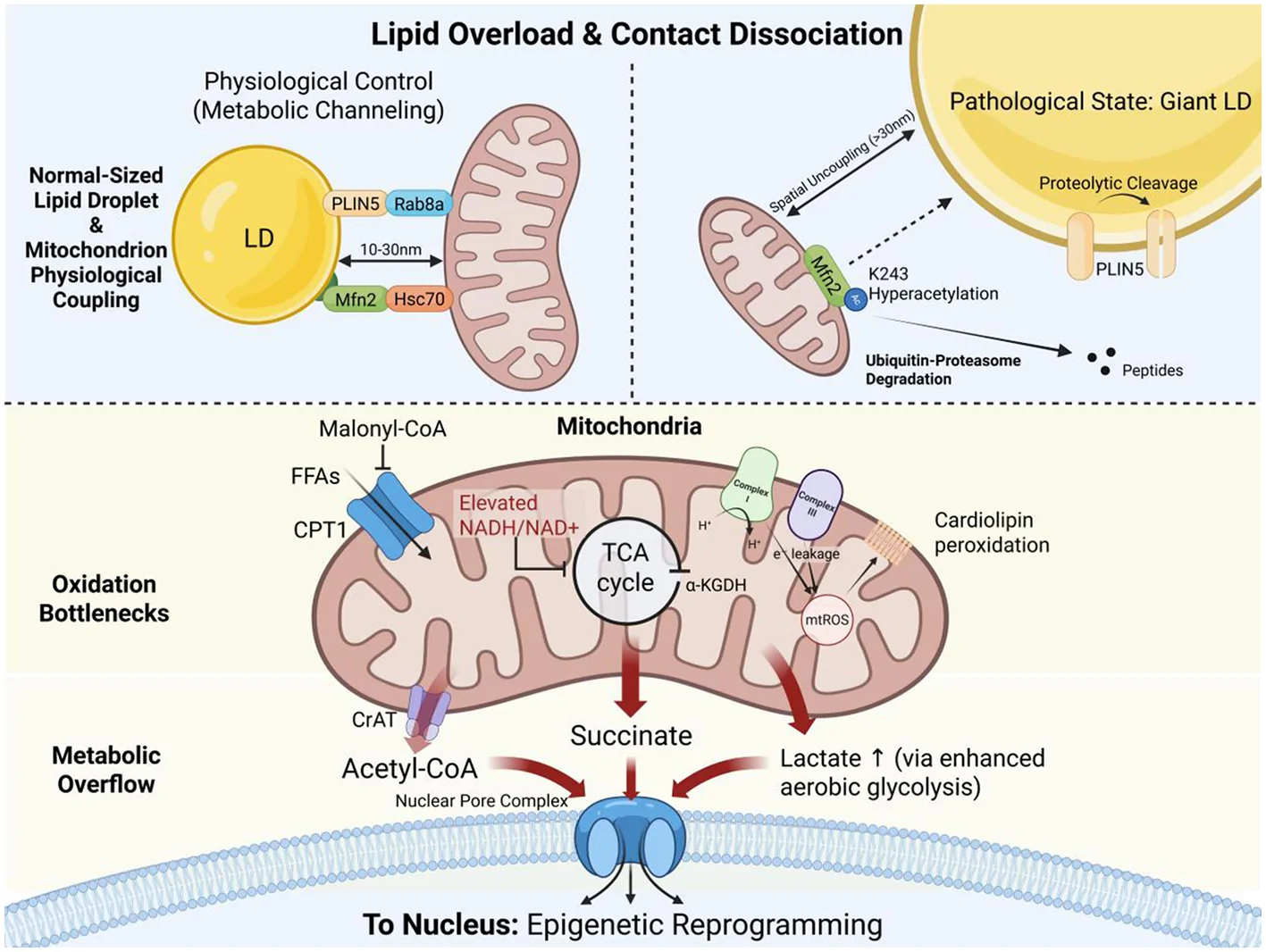
Lipid overload-induced organelle uncoupling and metabolic overflow. Chronic lipid overload disrupts skeletal muscle lipid droplet–mitochondria contact (LDMC) sites. This is marked by Mfn2 hyperacetylation at Lys243 and later ubiquitin-proteasome degradation, along with proteolytic cleavage of PLIN5. This causes spatial uncoupling (gap >30 nm). This structural separation disrupts efficient metabolic channeling, causing an accumulation of cytosolic lipid intermediates. In the mitochondria, too much FFA influx and de novo lipid synthesis increase malonyl-CoA, which blocks CPT1 and limits long-chain fatty acyl-CoA from entering the matrix. The resulting substrate overload and high NADH/NAD^+^ ratio lower the activity of TCA cycle dehydrogenases, like α-KGDH. As a result, unoxidized substrates and metabolic intermediates, like Acetyl-CoA (transported through the carnitine acetyltransferase, CrAT, shuttle), succinate, and lactate (coming from higher aerobic glycolysis), overflow from the mitochondrial matrix and cytosol into the nucleus to help epigenetic reprogramming. At the same time, hyper-reduction of the ubiquinone pool causes electron leakage from Complexes I and III, making mitochondrial reactive oxygen species (mtROS) and increasing cardiolipin peroxidation.

## 4 Metabolite-driven epigenetic reprogramming in the nucleus

The nucleocytoplasmic transport of polar metabolic intermediates connects mitochondrial metabolism to transcriptional regulation. By acting as covalent carbon donors or competitive inhibitors of specific dioxygenases, these metabolites control nuclear enzyme activity based on mitochondrial redox changes. Especially, the accumulation of Acetyl-CoA, succinate, and lactate is linked to a change of local chromatin from a repressive state to an accessible state, which changes the structural barriers that regulate atrophy-associated genes.

### 4.1 Metabolites as epigenetic substrates and enzymatic modulators

The changed nucleocytoplasmic distribution of energy intermediates affects the activity of histone-modifying enzymes. In the nucleus, the rate of epigenetic modification is linked to substrate availability; for example, *in vitro* cell-free assays show that histone acetyltransferases (HATs) show Michaelis constant (*Km*) values ranging from 1 to 10 μM, a range that matches the basal nuclear concentration of Acetyl-CoA (69, 70). As a result, high nuclear concentrations of Acetyl-CoA, succinate, and lactate during lipid stress control the activity of specific epigenetic modifying enzymes (71).

Acetyl-CoA acts as the acetyl donor for HAT families, like p300/CBP, GNAT, and MYST, which transfer acetyl groups to specific histone lysine residues (69, 70). The covalent addition of an acetyl group neutralizes the positive charge of the ϵ-amino group on histone tails, which lowers the electrostatic attraction between the histone octamer and the negatively charged DNA phosphate backbone (72). This electrostatic change causes nucleosome decompaction and steric relaxation of chromatin arrays, which increases the accessibility of specific loci for transcriptional machinery (73).

At the same time, succinate acts as a competitive inhibitor of α-ketoglutarate (α-KG)-dependent dioxygenases, a superfamily that includes TET proteins (DNA demethylation) and JmjC-domain enzymes (histone demethylation) (74). Succinate shares stereochemical structural features with α-KG but lacks the α-keto group needed for oxidative decarboxylation. High nuclear succinate levels competitively block these dioxygenases (60). Kinetic tests show that succinate blocks JmjC family enzymes with a *Ki* of 350–500 μM (60), a concentration limit that is expected to be reached in the nucleoplasm during severe intracellular lipid accumulation. This active-site occupancy limits the formation of the oxoiron(IV) intermediate, lowering DNA and histone demethylation rates (60). This targeted blocking of H3K27-specific demethylases (KDM6A/JMJD3) keeps DNA hypermethylation and keeps repressive H3K27me3 marks at bivalent promoters, sterically locking these responsive loci in a transcriptionally inactive state (75).

In the context of changed glycolysis, high lactate concentrations give a carbon source for histone lactylation (H3K18la and H3K9la) (65, 68). This covalent modification, catalyzed by p300/CBP using lactyl-CoA, brings a larger steric volume and higher polarity that changes nucleosomal stability (76, 77). Having acetylation, lactylation, and succinylation at the same time creates a substrate-dependent metabolic-epigenetic interface (78). The local deposition of these modifications—like KAT2A and α-KGDH-mediated histone succinylation—connects local chromatin structural states directly to mitochondrial metabolic output (79). At the same time, the drop in the nuclear NAD^+^ pool lowers the catalytic activity of NAD+-dependent deacylases (SIRT1/SIRT5), which limits the hydrolytic removal of these acyl modifications (80, 81). As a result, this combined failure of deacylation and metabolite-driven hyperacylation helps the transcriptional activation of atrophy-associated genes.

### 4.2 Imbalance of epigenetic “writers” and “erasers” under metabolic stress

The metabolic microenvironment changes the activity of nuclear epigenetic regulators, which shifts the functional balance between “writers” (acyltransferases/methyltransferases) and “erasers” (deacylases/demethylases). This enzymatic change is caused by local substrate availability and cofactor depletion.

Especially, *DNMT3B* expression rises during lipid exposure, helping non-CpG hypermethylation at the *PGC-1α* promoter and lowering mitochondrial biogenesis (17). At the same time, the long-term structural separation of LDMC sites uses up the available PLIN5 pool, blocking its stress-responsive nuclear translocation. The lack of this structural co-regulator in the nucleoplasm blocks SIRT1 activation, which shifts the balance toward GCN5-mediated PGC-1α hyperacetylation and transcriptional suppression (18, 19).

The regulatory capacity of “eraser” enzymes shows class-specific differences across the mitochondrial-nuclear axis. Histone lactylation rises at the same time as the blocking of Class I HDACs and the Sirtuin family, which have built-in delactylase activity (80). *In vitro* biochemical tests show that lactate blocks HDAC1–3 with an *IC50* of about 40 mM, a high concentration limit that may be reached in compartmentalized subcellular microdomains during severe glycolytic shifts (82). Also, the drop of the nuclear NAD^+^ pool during lipid stress directly blocks NAD+-dependent Class III deacylases, like SIRT5 and SIRT1 (81). This failure in active deacylation machinery causes the ongoing accumulation of acyl-lysine marks in the nucleus, strongly connecting mitochondrial metabolic stalling to epigenetic modifications (81).

On the other hand, zinc-dependent histone deacetylases show functional redirection instead of total blocking. Metabolic stress causes the nucleocytoplasmic shuttling of Class IIa HDACs (HDAC4/5), sending them to block the *Dach2* locus, an event needed to free transcriptional activators for E3 ligase promoters (83). At the same time, Class IV HDAC11 shifts its affinity toward myogenic differentiation loci to remove H3K9ac and H4K16ac marks, sterically locking these specific chromatin regions in a closed state (84). Meanwhile, Class I HDAC1 targets non-histone proteins, especially changing acetylation marks on FoxO3a to expose its nuclear localization signal (NLS), ensuring its constant import to keep atrophy-associated gene expression (85). This changed balance shifts the epigenetic landscape: global deacylation is blocked, while specific HDAC redirection and high metabolite substrates together prepare chromatin regions linked to muscle atrophy.

### 4.3 Chromatin accessibility and the transcriptional release of atrophic genes

This metabolic-epigenetic cascade ends in structural chromatin remodeling at muscle-specific loci (21). The accumulation of activation marks, like H3K27ac, H3K9ac, and acyl-lysine modifications, causes nucleosomal decompaction. This changes local chromatin into a transcriptionally permissive state (78, 86). This targeted structural relaxation allows the physical binding of transcription factors (87).

The spatial modification of these regulatory elements allows the recruitment of FoxO and Smad families (88). At the same time, the drop in repressive marks, like H3K9me2, is linked to the separation of heterochromatin protein 1 (HP1) clusters from these target promoters (88). With past steric barriers removed, FoxO3a and Smad2/3 successfully gather on Forkhead Response Elements (FHREs) and Smad-binding motifs in the promoters of E3 ubiquitin ligases, especially *Atrogin-1 (Fbxo32)* and *MuRF1 (Trim63)* (20, 89).

This promoter assembly acts as the direct downstream result of the nuclear signaling shifts mentioned before. Especially, the earlier HDAC4/5-mediated repression of *Dach2* removes downstream inhibitory limits, increasing the recruitment of Myogenin (*MyoG*) and needed co-activators directly to these E3 ligase promoters (83). At the same time, the HDAC1-modified FoxO3a, having exposed its NLS, successfully finishes nuclear import to carry out this targeted transcriptional activation (85).

Preclinical murine chromatin immunoprecipitation sequencing (ChIP-seq) data structurally map this binding, showing that FoxO3a occupies the FHRE located between -1.2 kb and -0.8 kb upstream of the *Atrogin-1* transcription start site (TSS) (89). In these exact genomic coordinates, metabolite-associated H3K27ac enrichment changes local chromatin accessibility. Similar regulatory patterns are seen at the *MuRF1* locus, where FoxO3a and Smad2/3 gather together in a region located -500 bp to -250 bp upstream of the *MuRF1* TSS (90). This clear chromatin remodeling helps pre-initiation complex assembly, greatly increasing steady-state mRNA transcription for *Atrogin-1* and MuRF1 (90). The irreversible change from sub-organellar metabolite overflow to an open chromatin state finally controls the execution of the muscle-wasting transcriptional program.

## 5 Transcriptional release of the atrophic program and muscle phenotype formation

### 5.1 The epigenetic-transcriptional nexus: from metabolic sensitization to atrophic priming

The change from metabolic stress to structural muscle wasting is linked to changes in the nuclear epigenetic landscape (88). After the earlier breakdown of organelle contact networks and the later nucleocytoplasmic overflow of metabolic intermediates (27, 53), this accumulation directly controls the activity of epigenetic-modifying enzymes (91).

Especially, the combined action of succinate-mediated demethylase blocking and acyl-CoA-driven hyperacylation (60, 65) acts as a core regulatory part of skeletal muscle proteostatic changes, creating a state of lasting epigenetic priming (88). The local deposition of activating histone marks, especially H3K27ac and H3K9la, along with the keeping of repressive marks like H3K9me2, completely changes chromatin architecture (68, 88). This setup makes sure that regulatory elements at the promoters of muscle-specific E3 ubiquitin ligases show higher accessibility, while myogenic and regenerative loci are kept in a transcriptionally restricted state (87).

Importantly, this epigenetic change controls the transcriptional output of the myofiber by changing its sensitivity to cytosolic signaling. Preclinical *in vitro* models studying “metabolic memory” show that lasting covalent epigenetic marks keep atrophic gene promoters in an open state even after stressor removal (92). This ongoing chromatin state is linked to a long catabolic trajectory in specific *in vitro* myoblast models (93). By lowering the activation limit for ubiquitin-proteasome degradation and shifting the balance away from protein synthesis and regeneration, this nucleocytoplasmic spread of metabolic stress creates the needed epigenetic base for the sarcopenic phenotype (88).

### 5.2 Epigenetic dysregulation of the proteostasis network

#### 5.2.1 Epigenetic release of the ubiquitin-proteasome system

The transcriptional activation of muscle-specific E3 ubiquitin ligases, *Atrogin-1/MAFbx (Fbxo32)* and *MuRF1 (Trim63)*, starts the ubiquitin-proteasome system (UPS) during skeletal muscle atrophy (90). Epigenetic changes at these loci help pre-initiation complex assembly, which causes higher mRNA transcripts for *Atrogin-1* and *MuRF1* (90). As shown before, taking away inhibitory acetylation marks from FoxO3a by Class I HDACs helps its nuclear import (85). Concurrently, the repression of *Dach2* by Class IIa HDACs enhances Myogenin recruitment to these promoters (83). Furthermore, Smad3 enhances FoxO3-mediated MuRF1 promoter activity in a DNA-binding-dependent manner (20). Upon translation, these ligases catalyze K^48^-linked polyubiquitination of sarcomeric contractile proteins, including myosin heavy chain (MyHC) and titin, targeting them to the 26S proteasome for degradation (94). *MuRF1* targets structural components of the contractile apparatus, whereas *Atrogin-1* regulates the degradation of protein synthesis initiation factors (94). Due to sustained chromatin accessibility, this targeted transcriptional activation maintains the UPS as a catabolic driver of myofibrillar protein degradation.

#### 5.2.2 Dual epigenetic-cytosolic blockade of the PI3K/Akt/mTOR axis

Lipotoxic skeletal muscle atrophy is accompanied by the profound suppression of anabolic capacity (10, 95). This reduction in protein synthesis is mediated by the signaling alteration of the PI3K/Akt/mTOR axis (96, 97). This impairment involves a synergistic combination of epigenetic modifications at growth factor loci and the downstream inhibition of cytosolic kinases (10, 88). In the nucleus, chronic metabolic stress alters DNA methylation patterns at the promoters of upstream anabolic effectors, such as insulin-like growth factor 1 (IGF-1), reducing basal transcriptional activation (88, 98).

Concurrently in the cytosol, the reduction of Akt signaling downregulates mTORC1-mediated translational initiation and alters the phosphorylation status of FoxO transcription factors (89). Membrane-localized DAG activates PKCθ, which catalyzes the inhibitory serine phosphorylation of IRS-1 at Ser1101 (99, 100). This modification prevents the tyrosine phosphorylation of IRS-1 needed for PI3K activation, causing structural insulin resistance (100). At the same time, subsarcolemmal ceramides increase Akt dephosphorylation through protein phosphatase 2A (PP2A) and limit Akt recruitment to the sarcolemma through atypical PKC isoforms, strongly setting up a changed regulation of the insulin signaling cascade (101).

This upstream blockade spreads directly to the mTORC1 complex. Intracellular accumulation of ceramides and DAGs changes the activation status of the Rag GTPase complex, which limits the lysosomal recruitment and activation of mTORC1 (102). This limit keeps eukaryotic translation initiation factor 4E-binding protein 1 (4E-BP1) in a hypophosphorylated state at residues Thr37/46 (102). As a result, 4E-BP1 stays tightly bound to eukaryotic initiation factor 4E (eIF4E), greatly lowering the cap-dependent translation of high-molecular-weight structural proteins, like myosin and titin (102). Experimental data show that this signaling change leads to a weaker protein synthetic response to amino acid availability and insulin stimulation (103).

#### 5.2.3 Defective autophagic flux and proteostatic collapse

The breakdown of proteostasis is made worse by changes in the autophagy-lysosome system (ALS), which is first controlled by FoxO3a-dependent transcriptional programs to clear uncoupled mitochondria (89).

In long-term lipotoxic conditions, this autophagic response is greatly changed, causing a huge drop in autophagic flux (104). Although autophagosome formation happens, the accumulation of lipids and oxidized lipoproteins lowers lysosomal hydrolytic capacity, matching lipid-induced lysosomal dysfunction (105, 106). This flux drop stops the clearance of autolysosomes, causing the toxic intracellular accumulation of undegraded organelles and lipofuscin (107, 108). The physical presence of these autophagic vacuoles mechanically changes the organization of the sarcomeric apparatus and is linked to ongoing oxidative stress (108).

Finally, the combined activation of UPS-mediated degradation, epigenetic suppression of anabolic transcripts, and ALS stopping greatly raises the fractional breakdown rate compared to the fractional synthetic rate (109). When catabolic flux is more than the regenerative capacity of the myofiber, ongoing sarcomeric protein depletion happens. Shown by preclinical models and clinical cohorts, this imbalance shows up structurally as a drop in muscle cross-sectional area (CSA) (109, 110). The combined activation of the UPS and autophagic systems is the final metabolic-epigenetic signaling output, locking the tissue into chronic muscle mass loss (102).

### 5.3 Epigenetic silencing of satellite cells and regenerative failure

Skeletal muscle homeostasis depends on the organized activation, proliferation, and differentiation of PAX7+ satellite cells controlled by myogenic factors (*Myf5*, *MyoD*, *MyoG*) (88). In lipotoxic microenvironments, this regenerative axis is badly damaged. This impairment features an epigenetic paradox: metabolic stress activates atrophic loci (20, 88) while transcriptionally repressing myogenic networks including *MyoD*, *MEF2C*, and *MyoG* (84, 88). As a result, regenerative failure adds to structural loss caused by UPS-mediated protein degradation (94) and Akt/mTOR-dependent translational suppression (102, 103), steadily speeding up muscle mass reduction by limiting myonuclear accretion.

#### 5.3.1 PAX7+ satellite-cell depletion and decline in regenerative reserve

PAX7+ satellite cells provide the essential cellular reserve for myogenic commitment. In preclinical models, this stem cell pool is continuously exposed to inflammatory mediators, including TNF-α and IL-1β (87). At the same time, mitochondrial substrate accumulation and high mtROS production cause lipid peroxidation and stress-kinase activation (111). These niche stressors change the physiological balance controlling satellite-cell quiescence and activation. Also, intrinsic epigenetic-metabolic factors control stem-cell fate; for example, SIRT2 activity changes Nur77 deacetylation, directly affecting *Myf5* transcriptional output and PAX7+ stem-cell maintenance (112). These observations show that satellite cell depletion is caused by combined intrinsic and microenvironmental epigenetic changes.

#### 5.3.2 Myf5/MyoD imbalance and impaired myogenic commitment

After activation, satellite cells need ongoing *Myf5* and *MyoD* expression for lineage commitment and cell-cycle progression. Lipotoxic metabolic stress changes chromatin architecture to suppress this network. The histone lysine methyltransferase SUV39H1, working with DNA methylation pathways, adds repressive H3K9me2 marks at the *MyoD* promoter, helping heterochromatin protein 1 (HP1) recruitment and stopping *MyoD* transcription (88). This epigenetic block limits the transition of activated satellite cells into downstream differentiation programs. At the same time, the deacetylation of differentiation-related loci by HDAC11 blocks progression (84). By directly controlling *Myf5* transcriptional output, these metabolic-epigenetic problems lower overall regenerative potential (112), shifting the balance from muscle repair toward atrophic priming (20, 88).

#### 5.3.3 Suppression of the MyoD/MyoG/MEF2C axis and failure of terminal differentiation

Terminal differentiation and myotube fusion rely upon the execution of the *MyoG* and *MEF2C* transcriptional programs. Metabolic sensing pathways direct the Class IV deacetylase HDAC11 to differentiation gene regulatory regions, where it removes active H3K9ac and H4K16ac marks (84). This enzymatic erasure decreases chromatin accessibility, restricting transcriptional machinery access and suppressing *MEF2C* and *MyoG* expression (84, 88). While *MyoG* primarily drives terminal differentiation, stress-induced nucleocytoplasmic shifts in Class IIa HDACs repress *Dach2*, actively redirecting *MyoG* from myogenic loci to the promoters of E3 ubiquitin ligases (83). This pathological recruitment exemplifies the profound transcriptional reprogramming induced by lipotoxicity.

#### 5.3.4 Integration of regenerative failure with the sarcopenic phenotype

Weakened muscle repair is the final cellular result of satellite-cell exhaustion and myogenic silencing. The lipotoxic niche imposes concurrent pathogenic pressures: inflammatory signaling (TNF-α, IL-1β) (87) and lipid peroxidation byproducts (MDA, 4-HNE) activate stress cascades such as JNK and NF-κB (111), subverting reparative differentiation. At the same time, lipid-induced changes keep 4E-BP1 in a hypophosphorylated state to block eIF4F complex formation (102), which weakens the protein synthetic response to amino acid availability (103). Added to this metabolic suppression is clear epigenetic remodeling: HP1 recruitment at the *MyoD* promoter (88) and HDAC11-mediated active mark erasure (84) stop repair gene transcription, while *Atrogin-1* and *MuRF1* go through constant chromatin de-repression (20, 88).

The combination of inflammatory signaling, translational suppression, and epigenetic changes is linked to lower myofiber recovery in models of lipotoxic sarcopenia. Muscle atrophy is linked to higher protein degradation (94), blocked translational initiation and amino acid responsiveness, and the breakdown of the *Myf5*/*MyoD*/*MyoG* regenerative network (112). In this environment, regulatory mechanisms are changed: the catabolic arm is turned on through FoxO/Smad pathways, the anabolic arm is affected by IRS-1/PI3K/Akt/mTOR blocking, and the regenerative arm is changed through the epigenetic repression of myogenic programs. The nuclear presence of metabolic intermediates is linked to this balance, helping the expression of *Atrogin-1* and *MuRF1* (20, 88) while lowering *MyoD*, *MyoG*, and *MEF2C* (84), and dropping the PAX7+ cell pool (112). These combined changes lead to the drop in myofiber cross-sectional area and muscle mass (110).

### 5.4 Dimensionality of the sarcopenic phenotype: from molecular signatures to tissue architecture

Epigenetic modifications, altered protein degradation, and regenerative failure work together to cause the sarcopenic phenotype (88). This complex structural decline happens at the molecular, histological, and functional levels, and it directly shows upstream metabolic adaptations (10).

At the molecular and cellular level, UPS-mediated proteolysis targets very specific sarcomeric substrates (113). E3 ubiquitin ligases, especially *MuRF1*, recognize and polyubiquitinate core structural components including myosin heavy chain (MyHC) and titin, and send them for 26S proteasomal degradation (114, 115). This targeted myofibrillar depletion greatly weakens the mechanical integrity and force-generating capacity of the myofiber (116).

Histologically, this atrophic remodeling is very specific to the fiber type (117). Chronic lipotoxic conditions cause more atrophy and cross-sectional area reduction in fast-twitch glycolytic (Type II) fibers than in slow-twitch oxidative (Type I) fibers (118). This fiber-type-specific vulnerability is linked to the localized accumulation of metabolic intermediates, such as lactate, which keep up epigenetic gene repression (119). At the same time, abnormal intramyocellular lipid handling and lipotoxicity-induced cellular stress deeply change the interstitial space (120). This pathological niche recruits fibro-adipogenic progenitors (FAPs), leading to severe lipid infiltration defined as myosteatosis (121). Myosteatosis physically disturbs the alignment of adjacent myofibers and breaks the architectural matrix of the tissue (122).

Functionally, sarcomeric depletion and histological deterioration determine overall muscle performance (122). The degradation of MyHC and titin directly reduces specific tension, which is the maximal contractile force generated per unit of muscle cross-sectional area (CSA) (108). In addition, the interstitial expansion of adipose tissue disrupts lateral and longitudinal force transmission efficiency along the muscle axis (121). Combined with mitochondrial uncoupling and oxidative stress, these structural defects greatly lower muscle fatigue thresholds (123). Together, these multi-dimensional structural and functional impairments cause the clinical manifestations of T2DM-associated sarcopenia (124).

Finally, the pathophysiology of T2DM-associated sarcopenia is controlled by the strict coordination of the metabolic-epigenetic axis (88). The lipotoxic microenvironment leads to a progressive chain of events: subcellular organelle uncoupling causes metabolite overflow, which drives the chromatin remodeling needed to activate the UPS, suppress the PI3K/Akt/mTOR pathway, and silence the regenerative satellite-cell reserve (125, 126). Because this atrophic program is fixed at the chromatin level (127), effective therapeutic interventions must use multiple methods—restoring subcellular contact topology and resetting the pathological epigenetic landscape at the same time (128) (**Figure 3**).

**Figure 3:**
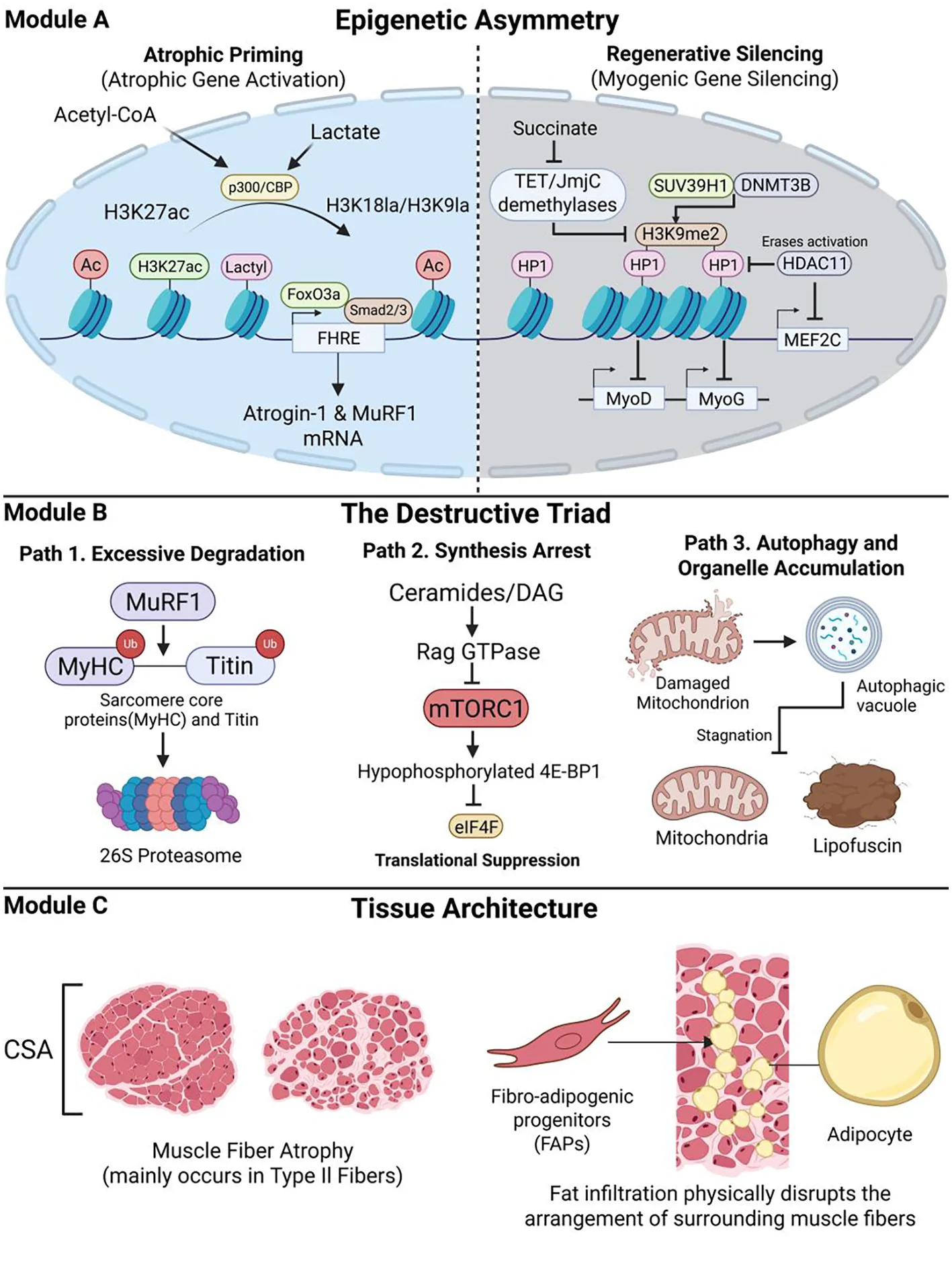
Metabolic-epigenetic axis driving transcriptional release and tissue-level atrophy. (Module A) Epigenetic asymmetry in the nucleus: Acetyl-CoA and lactate accumulate, and they promote histone acetylation (H3K27ac) and lactylation (H3K18la/H3K9la) via p300/CBP. This helps FoxO3a/Smad2/3 bind to FHREs and activate atrophic genes (Atrogin-1 and MuRF1). On the other hand, succinate inhibits TET/ JmjC-domain demethylases. This inhibition, together with SUV39H1 and DNMTs, promotes H3K9me2 accumulation and HP1 recruitment. At the same time, HDAC11 removes active marks (H3K9ac/H4K16ac), and together they repress myogenic factors (MyoD, MyoG, MEF2C). (Module B) The destructive triad of proteostatic collapse: Path 1 involves excessive UPS-mediated degradation of sarcomeric proteins (MyHC and titin) via MuRF1. Path 2 shows the inhibition of mTORC1 (via Rag GTPase) and later 4E-BP1 hypophosphorylation, which limits eIF4F assembly and stops anabolic translation. Path 3 shows autophagic flux stagnation, causing the accumulation of damaged mitochondria and lipofuscin. (Module C) Tissue architecture alterations: Lipotoxicity leads to reduced muscle fiber cross-sectional area (CSA), especially in Type II fibers, together with interstitial fat infiltration (myosteatosis) mediated by fibro-adipogenic progenitors (FAPs). This physically disrupts muscle fiber alignment.

## 6 Multimodal therapeutic strategies for T2DM-associated sarcopenia

Effective therapeutic strategies for T2DM-associated sarcopenia must move from targeting single downstream kinases to a combined approach that addresses three sequential pathogenic tiers (1): structural topological rescue (2), metabolic intermediate clearance, and (3) epigenetic resetting. Because single treatments, like isolated epigenetic blockade, are often bypassed by continuous upstream metabolic flux, long-term success requires combining these sub-cellular targets into synergistic treatments.

### 6.1 Restoring subcellular architecture: topographical rescues at membrane contact sites

Because the structural separation of organellar contact zones causes metabolic overflow, therapies aimed at restoring spatial topology directly reduce the cytosolic accumulation of reactive intermediates at their source.

#### 6.1.1 Targeted amelioration of ER stress and modulation of MAMs calcium coupling

Strategies targeting MAMs focus on reducing endoplasmic reticulum (ER) stress at the subcellular membrane interface (129). In murine lipotoxic models, pharmacological chaperones like 4-PBA reduce the protein folding burden and restore the physical contact of the ER and outer mitochondrial membranes (130). These treatments stabilize the IP3R-GRP75-VDAC1 tethering complex, keeping directional calcium transfer. This restored calcium signaling maintains the catalytic activity of TCA cycle dehydrogenases, keeping up oxidative flux (131). In addition, keeping TCA cycle flux via MAMs stabilization theoretically reduces the upstream substrate pool for pathological histone modifications, although direct causality remains to be definitively established in skeletal muscle models (13).

But, applying this to human sarcopenia faces a narrow therapeutic window: although severe ER stress causes atrophy, basal unfolded protein response activity is needed for muscle proteostasis and exercise adaptation. Therefore, systemic UPR suppression may accidentally harm normal myofiber maintenance, which makes it necessary to develop skeletal muscle-targeted delivery systems.

#### 6.1.2 Exercise-induced organelle restructuring and PGC-1α4-mediated transcriptional protection

Mechanical loading provides a two-part defense against lipotoxicity via the AMPK/PGC-1α signaling axis. In the short term, physical training causes the upregulation of Mfn2 to spatially strengthen the LD-mitochondria (Mfn2-Hsc70) transmembrane interaction (132, 133). This structural support reduces the space between organelles, which helps localized fatty acid transport machinery and limits the cytosolic diffusion of ceramides and diacylglycerols.

In the long term, mechanical stress causes alternative splicing to produce the truncated PGC-1α4 isoform, which is very different from the oxidative function of full-length PGC-1α1 (134, 135). This parallel transcriptional change separates energetic regulation from protein turnover. PGC-1α4 directly blocks the myostatin-Smad axis and activates IGF-1 pathways. This stops E3 ubiquitin ligase transcription while the physical contact zones handle metabolic overflow (135). Finally, this combined action in space and time between physical organelle restructuring and isoform-specific transcriptional reprogramming acts as a main non-pharmacological defense against myofibrillar atrophy.

### 6.2 Alleviating metabolic overflow: precision targeting of mitochondrial bottlenecks

Mitochondrial metabolic stalling drives the nucleocytoplasmic efflux of polar intermediates (Acetyl-CoA, succinate, and lactate). Interventions must therefore bypass specific enzymatic bottlenecks to deplete the substrate pool fueling pathological epigenetic remodeling, though achieving subcellular precision remains challenging.

#### 6.2.1 The limits of systemic metabolic modulators

Systemic drugs, like SGLT2 inhibitors and GLP-1 receptor agonists, work well to reduce systemic glucolipotoxicity (136). But, clinical evidence shows that the large systemic caloric deficit caused by these drugs can accidentally cause lean mass loss along with fat reduction (137, 138). Crucially, the alleviation of systemic lipid burdens does not immediately guarantee the reversal of localized transcriptional pathologies. While recent evidence, such as the study by Hinte et al. (139), demonstrates that adipose tissue retains a robust epigenetic memory of obesity following systemic weight loss, analogous mechanisms exist in skeletal muscle. Both *in vitro* models and human cohorts confirm that skeletal muscle independently retains long-lasting epigenetic “memory” following initial stress exposure (93, 127). Consequently, the upstream removal of systemic lipid overload does not synchronously erase the established downstream chromatin modifications. Because these covalent histone acylations are biochemically stable, they persist even after the primary metabolic stress is alleviated. This persistent epigenetic state explains why systemic metabolic improvement alone is insufficient for complete local recovery, highlighting the necessity for muscle-directed, organelle-specific interventions (10).

#### 6.2.2 Pharmacological rerouting: opportunities and mechanistic paradoxes

Preclinical strategies try to directly redirect metabolic flux to bypass localized bottlenecks (10). For example, exogenous octanoic acid (a medium-chain fatty acid, MCFA) moves directly into the mitochondrial matrix, bypassing the malonyl-CoA-inhibited carnitine shuttle (CPT1) to restart β-oxidation (140). But, this method has a mechanistic paradox: if the downstream TCA cycle and respiratory chain remain stalled by a high ratio, forcing MCFA oxidation will actually worsen the matrix Acetyl-CoA buildup, feeding instead of reversing epigenetic hyperacetylation (47, 141).

At the same time at the cytosolic level, proof-of-concept probes like the pleiotropic polyphenol gallic acid have been used *in vitro* to inhibit lactate dehydrogenase A (LDHA) (63). By limiting the pyruvate-to-lactate conversion, this method empties the nuclear lactyl-CoA pool needed by p300/CBP for histone lactylation (142, 143). Still, turning general enzymatic inhibitors into targeted therapies needs great care; blocking LDHA basically risks breaking normal glycolytic shifts in skeletal muscle and causing severe toxicity in obligate glycolytic cells, including erythrocytes (76).

### 6.3 Resetting the epigenetic landscape: terminal transcriptional blockade

Epigenetic homeostasis depends on the enzymatic balance between acyltransferases (“writers”) and deacylases/demethylases (“erasers”). Because the lipotoxic microenvironment causes structural chromatin remodeling through metabolite overflow, using drugs to change these nuclear enzymes offers a final block against atrophic transcriptional programs.

#### 6.3.1 Pharmacological targeting of acyltransferases and non-histone modifiers

The pathological buildup of histone lactylation and succinylation depends on abnormal acyltransferase activity (144). In preclinical models, molecular probes like the p300/CBP inhibitor C646 and KAT2A inhibitors limit the enzymatic transfer of acyl groups to histone tails, and this restricts chromatin accessibility at catabolic loci (145). At the same time, epigenetic modulators also target non-histone proteins: Specific Class I HDAC inhibitors, like MS-275, stop the deacetylation of FoxO3a (87). Instead of condensing chromatin, this specific change harms FoxO3a nuclear retention, weakening the ubiquitin-proteasome system (128). However, translating these findings remains challenging; drugs like C646 show significant pleiotropy and poor *in vivo* stability, and long-term systemic epigenetic interference risks severe off-target transcriptional silencing in non-muscle tissues (88).

#### 6.3.2 Reactivation of the epigenetic “erasers”: the NAD^+^ translational gap

In lipotoxic skeletal muscle, chronic oxidative stress overactivates poly(ADP-ribose) polymerases, depleting the nuclear NAD^+^ pool and severely suppressing NAD+-dependent epigenetic “erasers.” (128) Preclinical supplementation with NAD^+^ precursors, like nicotinamide riboside (NR) or nicotinamide mononucleotide (NMN), effectively refills the nuclear NAD^+^/NADH ratio (146). This availability reactivates nuclear SIRT1, which actively removes permissive acetylation marks from atrophic gene promoters and further limits FoxO3a transcriptional activity (147). Even with strong results in murine sarcopenia models, the therapeutic potential of NAD^+^ boosters faces a well-known translational gap; recent human clinical trials have shown mixed or small outcomes regarding lean mass preservation. This shows that restoring NAD^+^ alone might not be enough to fully reverse established lipotoxic muscle wasting in humans (148).

### 6.4 Comparative pharmacological profiling of targeted agents

Evaluating new treatments for T2DM-associated sarcopenia requires mapping their targets across sub-cellular compartments and separating their local muscular effects from systemic metabolic results (**Table 1**).

**Table 1:** Multidimensional pharmacological profiling of therapeutics in T2DM-associated sarcopenia.

Intervention strategy	Representative agent/stimulus	Subcellular localization	Primary target/mechanism	Epigenetic impact	Pleiotropy & systemic vs. muscle effects
Topological Rescue	Exercise/Mfn2 Activators	MAMs & LDMC Interface	Upregulates Mfn2; Restores <30nm organelle apposition	Restricts upstream metabolite supply for histone acylation	Highly muscle-specific; Improves systemic insulin sensitivity naturally
Topological Rescue	Pharmacological Chaperones (*e.g.*, PBA)	Endoplasmic Reticulum (ER)	Mitigates ER stress; Preserves IP3R-VDAC1 calcium flux	Indirect (prevents TCA cycle stalling and subsequent succinate overflow)	Poor tissue specificity; May disrupt physiological UPR in non-target tissues
Metabolic Bypass	Octanoic Acid (MCFAs)	Cytosol to Mitochondrial Matrix	Bypasses CPT1 bottleneck; Direct entry for β-oxidation	Depletes nuclear Acetyl-CoA supply by alleviating matrix backlog	May induce hepatic ketogenesis or systemic lipid profile shifts
Metabolic Bypass	Gallic Acid (LDHA inhibitor)	Cytosol	Inhibits pyruvate-to-lactate conversion	Depletes nuclear lactyl-CoA pool, reversing H3K9la/H3K18la	Potential impairment of physiological glycolytic shifts in erythrocytes or brain
Epigenetic ‘Eraser’	NR/NMN (NAD^+^ Precursors)	Nucleoplasm & Matrix	Replenishes NAD^+^ pool; Reactivates SIRT1/SIRT5	Restores active deacetylation and desuccinylation	Systemic benefits (anti-aging); Safe, but may have limited potency as a standalone monotherapy
Epigenetic ‘Writer’ Blockade	C646, KAT2A Inhibitors	Nucleoplasm	Competitively inhibits p300/CBP or KAT2A acyltransferase	Directly halts H3K27ac and H3 succinylation, condensing chromatin	High Pleiotropy: Systemic use risks severe off-target transcriptional silencing in immune/neural cells
Systemic Clearers	GLP-1 RAs, SGLT2i	Systemic (Receptor-mediated)	Reduces systemic glucolipotoxicity via central/renal targets	Unclear local epigenetic impact in myonuclei	Risk of Lean Mass Loss: Systemic caloric deficit may suppress muscle mTORC1 axis

This spatial profiling shows a basic pharmacological trade-off between upstream and downstream interventions (10). Upstream topological modulators, including potential Mfn2 activators, target the structural origin of metabolic overflow, but their clinical use is currently limited by a lack of muscle-specific delivery systems (149). On the other hand, downstream nuclear-targeted probes like C646 directly block atrophic chromatin remodeling but have poor *in vivo* stability and carry severe risks of pleiotropic off-target transcriptional silencing if given systemically (88, 128).Recognizing these precise pharmacokinetic and sub-cellular limitations—rather than relying on isolated mechanistic pathways—dictates the strict rationale for designing combinatorial therapeutic strategies (150) (**Figure 4**).

**Figure 4:**
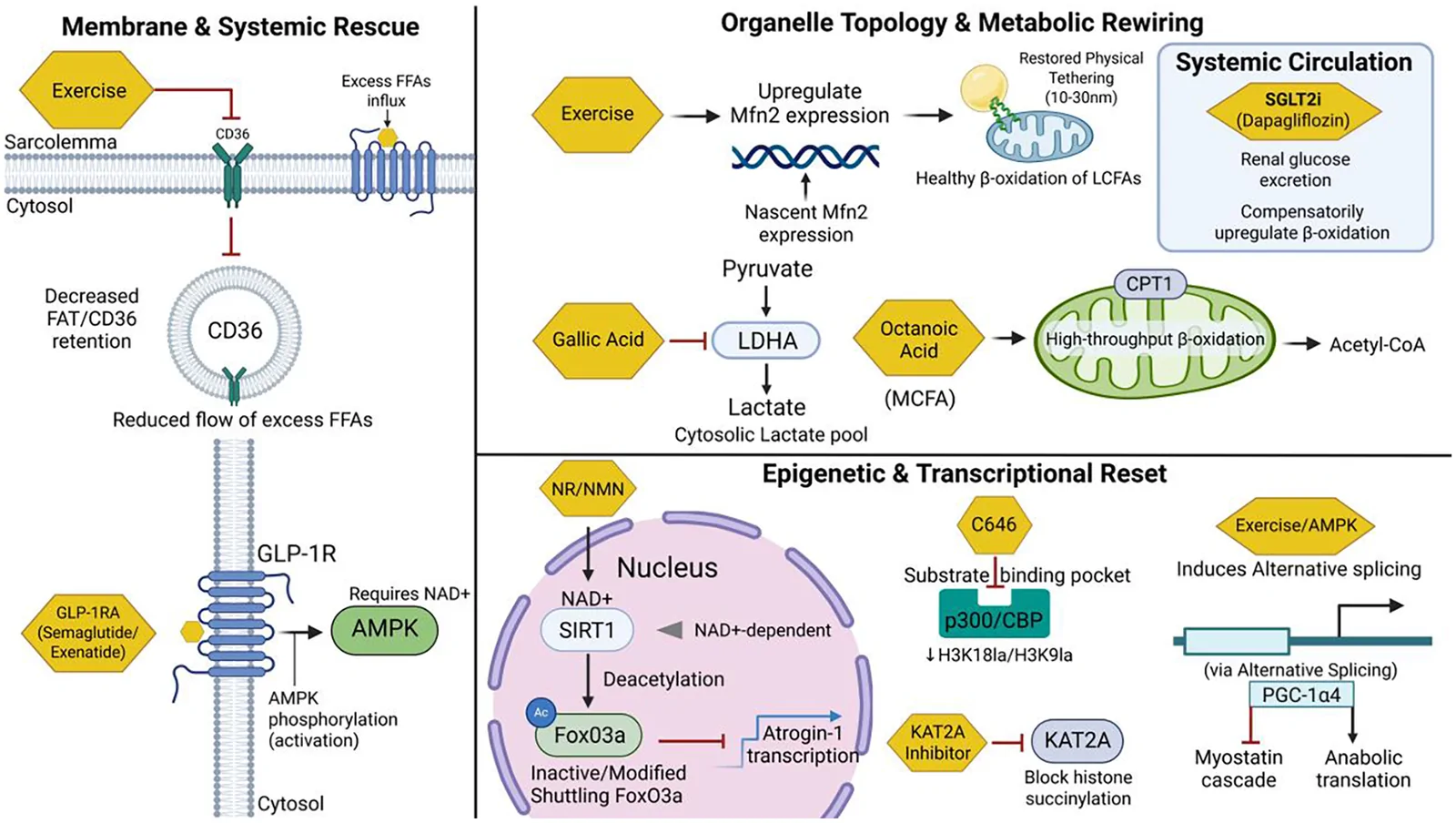
Multimodal therapeutic strategies for T2DM-associated sarcopenia. (Membrane & Systemic Rescue) Exercise and GLP-1 receptor agonists (GLP-1RAs) mitigate excess free fatty acid (FFA) influx by decreasing sarcolemmal FAT/CD36 retention. Additionally, GLP-1R activation stimulates downstream AMPK phosphorylation. (Organelle Topology & Metabolic Rewiring) Exercise upregulates nascent Mfn2 expression to restore physiological LDMC physical tethering (10–30 nm), thereby supporting the healthy β-oxidation of long-chain fatty acids (LCFAs). Within the systemic circulation, SGLT2 inhibitors (SGLT2i) promote renal glucose excretion, which compensatorily upregulates systemic β-oxidation. Concurrently, targeted pharmacological agents alleviate localized oxidation bottlenecks: gallic acid inhibits LDHA to decrease the cytosolic lactate pool, while octanoic acid (a mediumchain fatty acid, MCFA) bypasses CPT1 restriction to drive highthroughput b-oxidation. (Epigenetic & Transcriptional Reset) Replenishment of the nuclear NAD+ pool via NR/NMN supplementation reactivates SIRT1, facilitating the deacetylation of FoxO3a to suppress atrophic transcription. Epigenetic “writers” are restricted by specific inhibitors: C646 occupies the substrate binding pocket of p300/CBP to reduce histone lactylation (H3K18la/H3K9la), and KAT2A inhibitors block histone succinylation. Finally, exercise-induced AMPK activation promotes the alternative splicing of *PGC-1α* to the *PGC-1α4* isoform, which simultaneously represses the myostatin catabolic cascade and enhances anabolic translation.

### 6.5 Multimodal combination strategies: spatiotemporal synergy

Standalone interventions are mechanistically insufficient: physical topological repair is gradual and cannot acutely reverse established chromatin modifications, whereas isolated epigenetic inhibitors are transiently effective if upstream lipotoxic flux continues. Sustained efficacy requires spatiotemporal synergy between upstream and downstream targets.

#### 6.5.1 The “topological rescue + epigenetic reset” paradigm

A logical combination strategy combines the upstream restriction of metabolite overflow with downstream transcriptional resetting (10). For example, pairing mechanical loading (which structurally reconnects LDMC sites via the AMPK axis) with NAD^+^ precursors (NR/NMN) provides a helpful two-part mechanism (27, 151). Exercise reduces the continuous nucleocytoplasmic flux of acyl-CoAs, while the drug-restored NAD^+^ pool supports SIRT1-mediated deacetylation of atrophic promoters, especially *Atrogin-1* (128, 134). In theory, this combination secures the metabolic source at the organelle interface and at the same time removes existing pathological epigenetic marks inside the nucleus (150).

#### 6.5.2 Muscle-targeted delivery to offset systemic catabolism

To prevent the lean-mass loss linked to systemic metabolic clearers (SGLT2i and GLP-1RAs), future strategies must combine systemic energy management with muscle-directed epigenetic preservation (137). One possible way uses muscle-targeted delivery vectors—like aptamer- or peptide-conjugated nanoparticles—to selectively deliver small-molecule epigenetic modulators, especially KAT2A or p300 inhibitors, directly into myofibers (152). This method aims to allow systemic fat reduction via GLP-1RAs while locally protecting skeletal muscle chromatin from catabolic priming (153). But, applying such highly targeted delivery systems for a common metabolic disease faces substantial preclinical barriers. These include limited skeletal muscle tissue penetration, poorly understood long-term epigenetic toxicity, and the biological risk of artificially separating local muscle proteostasis from whole-body energy states (10).

## 7 Discussion and perspectives

### 7.1 Technical bottlenecks: resolving subcellular and syncytial heterogeneity

Proving a clear link between microenvironmental metabolite fluxes and epigenetic modifications requires fixing major method problems that hide the space and time details of skeletal muscle metabolism.

#### 7.1.1 The pitfall of bulk omics and metabolic flux heterogeneity

Current mechanistic models rely a lot on whole tissue mixtures, which fundamentally obscure subcellular metabolic differences. The “average” whole-cell measurement greatly weakens steep concentration gradients of Acetyl-CoA or lactate kept inside specific microdomains—especially the space between the LD-mitochondria interface and the nucleoplasm (154). Also, reactive intermediates like acyl-CoAs have very short lives and break down quickly during tissue lysis. This always skews absolute nuclear concentration estimates (13).

#### 7.1.2 Blind spots in the skeletal muscle syncytium

Skeletal muscle fibers are huge syncytia that hold thousands of myonuclei. Inside a single myofiber, myonuclei show clear anatomical specializations—like those at the neuromuscular junction, centralized nuclei, or subsarcolemmal nuclei. This exposes them to very different local metabolic fluxes and mechanical stresses. So, their local epigenetic microenvironments differ a lot (134).

Even though single-nucleus omics methods like snRNA-seq and NEAT-seq avoid mixing up cells, the needed physical separation permanently destroys this syncytial architecture. This makes it impossible to map “cross-nuclear transcriptional gradients” along the fiber. On the other hand, new spatial technologies—like Spatial-CUT&Tag cross-referenced with MALDI-MSI—keep structural coordinates (155, 156), but they have a major resolution mismatch. Current MALDI-MSI lacks the sub-cellular resolution needed to measure metabolites inside a single ~5 μm myonucleus. In addition, these static spatial pictures naturally fail to show the highly dynamic, real-time link between rapid metabolite overflow and later chromatin structural changes.

### 7.2 Bench-to-bedside translation: liquid biopsies and exercise mimetics

Turning these molecular insights into clinical management means moving from reactive treatments to early, mechanism-based strategies.

#### 7.2.1 Epigenetic signatures as non-invasive biomarkers

In the clinic, sarcopenia is usually diagnosed only after permanent cross-sectional area (CSA) loss has happened. Because metabolic-epigenetic reprogramming happens right before structural protein breakdown, specific chromatin changes have potential as predictive biomarkers. Because routine muscle biopsies are not practical for screening people without symptoms, future research must use liquid biopsies. Checking if circulating cell-free nucleosomes (cf-nucleosomes) in plasma show muscle-derived pathological marks, especially H3K9la lactylation (157) or abnormal succinylation, could give non-invasive “early warning signals” (13). Finding these systemic epigenetic signatures could group T2DM patients in the early stages of sarcopenia. This would define a key time window for treatment before major muscle fiber collapse.

#### 7.2.2 Modality-specific exercise signatures and the challenge of mimetics

Exercise is still the most effective clinical treatment against sarcopenia, well fighting the metabolic-epigenetic axis through mechanisms specific to the exercise type. Aerobic exercise mainly turns on the AMPK axis to improve matrix oxidative capacity and increase Mfn2. This structurally fixes LDMC tethers to limit upstream metabolite overflow (39). On the other hand, resistance training (mechanical loading) clearly causes the alternative splicing of *PGC-1α4*. This directly blocks catabolic transcriptional networks, especially the myostatin-Smad axis (134). Together, these exercise types naturally take away substrates from pathological acyltransferases, and at the same time they boost anti-atrophic transcription.

Knowing this two-part mechanism is very important for making “exercise mimetics” for weak patients who cannot exercise. The next group of drugs must try to copy both the topological rescue (aerobic-like) and the transcriptional reset (resistance-like). But, making compounds that achieve this dual intra-myocellular specificity concurrently is highly challenging. They must do this without causing the severe systemic pleiotropy and heart or liver toxicity often linked to broad AMPK or epigenetic agonists (158). This remains a huge drug barrier.

### 7.3 Spatiotemporal dynamics and systems biology modeling

To fully understand the metabolite-epigenetic axis over the next decade, the field must move from finding static links to defining dynamic cause and effect in space and time.

#### 7.3.1 Resolving kinetic asynchrony via live-cell biosensors and perturbation

Current epigenomic profiling platforms, like CUT&Tag, capture only static chromatin landscapes, but metabolic changes happen much faster (159). To close this time gap, the field needs to combine subcellularly targeted, genetically encoded biosensors inside 3D human skeletal muscle organoids and microfluidic systems. Even though doing live-cell metabolite sensing and single-locus chromatin conformational tracking at the same time is still limited by current light diffraction and signal-to-noise limits (160), it is necessary to combine dynamic metabolite tracking with direct spatial interventions—like optogenetic metabolite release (161) or pharmacological uncaging. Moving from just watching to actively changing the system is the needed step to prove the exact kinetic cause and effect between metabolic bursts and chromatin remodeling.

#### 7.3.2 Metabolite-epigenome-3D genome systems modeling

Beyond basic tracking, the next big step is systems biology. Long-term metabolic stress can cause chromatin to go through higher-order structural reorganizations, like the shifting of topologically associating domains (TADs) (162, 163). Making predictive “Metabolite-Epigenome-3D Genome” mathematical models could calculate the lipid flux thresholds and time lengths needed to push these chromatin conformations into self-sustaining, metastable catabolic states. Instead of defining an absolute “point of no return,” defining these biophysical thresholds will give important mechanistic insights. Finally, this computer system will show the best time windows for targeted drug treatments, aiming to reverse T2DM-associated sarcopenia before abnormal transcriptional networks become deeply fixed.

### 7.4 Conclusions

Lipotoxic skeletal muscle remodeling works through a connected process covering material flux, organellar topology, and chromatin state changes. The chain of events described in this review—moving from organelle contact failure to metabolite buildup and epigenetic reprogramming—adds to the standard model of muscle atrophy. Instead of looking at sarcopenia only through standard cytosolic kinase pathways, this model carefully combines these known signaling events with micro-substrate-driven epigenetic regulation.

At the center of this axis, mitochondrial matrix oxidative efficiency heavily depends on the intact structure of LDMC sites. The separation of this organellar interface stops substrate oxidation and forces the release of polar metabolic substrates (Acetyl-CoA, succinate, and lactate) into the nucleus and cytoplasm. The resulting buildup of these molecules inside the nucleoplasm controls the action of chromatin-modifying enzymes. This directly turns local metabolic concentration differences into continuous, but drug-targetable, transcriptional activation programs. Finally, using this sub-organellar, metabolic-epigenetic view is needed to find new treatments that protect skeletal muscle health under the growing global burden of metabolic disease.
